# Impact of body weight on the achievement of minimal disease activity in patients with rheumatic diseases: a systematic review and meta-analysis

**DOI:** 10.1186/s13075-016-1194-8

**Published:** 2016-12-13

**Authors:** Roberta Lupoli, Paolo Pizzicato, Antonella Scalera, Pasquale Ambrosino, Manuela Amato, Rosario Peluso, Matteo Nicola Dario Di Minno

**Affiliations:** 1 Department of Clinical Medicine and Surgery, Federico II University, Naples, Italy; 2 Centro Cardiologico Monzino, Istituto di Ricovero e Cura a Carattere Scientifico (IRCCS), Milan, Italy; 3 Division of Cardiology, Department of Advanced Biomedical Sciences, Federico II University, Via Sergio Pansini 5, 80131 Naples, Italy

**Keywords:** Psoriatic arthritis, Rheumatoid arthritis, Obesity

## Abstract

**Background:**

In this study, we evaluated the impact of obesity and/or overweight on the achievement of minimal disease activity (MDA) in patients with psoriatic arthritis (PsA) and patients with rheumatoid arthritis (RA) receiving an anti-rheumatic treatment. Obesity can be considered a low-grade, chronic systemic inflammatory disease and some studies suggested that obese patients with rheumatic diseases exhibit a lower rate of low disease activity achievement during treatment with anti-rheumatic drugs.

**Methods:**

A systematic search was performed in major electronic databases (PubMed, Web of Science, Scopus, Embase) to identify studies reporting MDA achievement in obese and/or overweight patients with RA or PsA and in normal-weight RA or PsA control subjects. Results were expressed as Odds Ratios (ORs) with pertinent 95% Confidence Intervals (95%CIs).

**Results:**

We included 17 studies (10 on RA and 7 on PsA) comprising a total of 6693 patients (1562 with PsA and 5131 with RA) in the analysis. The MDA achievement rate was significantly lower in obese patients than in normal-weight subjects (OR 0.447, 95% CI 0.346–0.577, *p* < 0.001, *I* ^2^ = 62.6%, *p* < 0.001). Similarly, overweight patients showed a significantly lower prevalence of MDA achievement than normal-weight subjects (OR 0.867, 95% CI 0.757–0.994, *p* = 0.041, *I* ^2^ = 64%, *p* = 0.007). Interestingly, the effect of obesity on MDA was confirmed when we separately analyzed data on patients with RA and patients with PsA. In contrast, when we evaluated the effect of overweight, our results were confirmed for PsA but not for RA. A meta-regression analysis showed that follow-up duration, age, male sex, and treatment duration are covariates significantly affecting the effect of obesity/overweight on MDA achievement.

**Conclusions:**

The results of our meta-analysis suggest that obesity and overweight reduce the chances to achieve MDA in patients with rheumatic diseases receiving treatment with traditional or biologic disease-modifying antirheumatic drugs.

**Electronic supplementary material:**

The online version of this article (doi:10.1186/s13075-016-1194-8) contains supplementary material, which is available to authorized users.

## Background

Rheumatoid arthritis (RA) is a chronic inflammatory disease characterized by inflammation of the synovial tissue that leads to bone erosion and progressive disability. Its prevalence of 0.5–1% in the general population makes it the most common chronic inflammatory condition [1]. Psoriatic arthritis (PsA) is a chronic inflammatory joint disease affecting up to 40% of patient with psoriasis, with a prevalence of 0.3–1% in the general population and leading to severe physical limitations and disabilities [2].

Besides articular manifestations, PsA and RA are characterized by an increased cardiovascular (CV) risk [3, 4], as represented by a higher prevalence of metabolic syndrome and of some of its major features (obesity, hypertension, hypercholesterolemia, hypertriglyceridemia, impaired fasting glucose) [5], increased platelet reactivity [6–8], higher degree of subclinical atherosclerosis [4, 9], impairment of endothelial function, and arterial stiffness [10–12]. In particular, several studies have shown a higher prevalence of obesity in patient with PsA and patients with RA than in the general population. Obesity causes an abnormal expression of adipokines (e.g., tumor necrosis factor-α [TNF-α], interleukin-6 [IL-6], adiponectin, leptin), which leads to a proinflammatory status. Thus, obesity can be considered a low-grade, chronic systemic inflammatory disease [13, 14]. Moreover, immune-mediated inflammation may act synergistically with obesity-mediated inflammatory status and may influence disease activity in rheumatic diseases [13, 15, 16]. Some studies [17] suggested that obese patients with rheumatic diseases exhibit a lower rate of low disease activity achievement during treatment with antirheumatic drugs. However, these results were challenged and not confirmed by other studies [18, 19]. In the present meta-analysis, we evaluated whether obesity and overweight impact the clinical response in patients with PsA and patients with RA receiving treatment with traditional disease-modifying antirheumatic drugs (DMARDs) or biologic DMARDs.

## Methods

A protocol for this review was prospectively developed, detailing the specific objectives, the criteria for study selection, the approach to assessment of study quality, the outcomes, and the statistical methods.

### Search strategy

To identify all available studies, a detailed search of studies reporting the achievement of minimal disease activity (MDA) in obese/overweight patients with RA/PsA and in normal-weight control subjects was conducted according to Preferred Reporting Items for Systematic reviews and Meta-Analyses (PRISMA) guidelines [20]. A systematic search was performed in major electronic databases (PubMed, Web of Science, Scopus, Embase), using the following search terms in all possible combinations: *psoriatic arthritis, rheumatoid arthritis, obese, obesity, overweight, body mass index, body composition, body weight, adiposity.* The latest search was performed in June 2016. The search strategy was developed without any language or publication year restriction. In addition, the reference lists of all retrieved articles were manually reviewed. In case of missing data, study authors were contacted by email to try to retrieve original data. Two independent authors (RL and PP) analyzed each article and performed the data extraction independently. In case of disagreement, a third investigator was consulted (MNDDM). Discrepancies were resolved by consensus. Selection results are reported according to a PRISMA flowchart (Additional file 1).

### Data extraction and quality assessment

According to the prespecified protocol, all studies comparing the rate of MDA achievement in obese or overweight patients with RA or PsA and in normal-weight control subjects were included. Case reports, animal models, and reviews were excluded. To be included in the analysis, a study had to provide the rate of MDA achievement in obese and/or in overweight patients with RA and/or PsA versus normal-weight control subjects. Also, studies reporting the OR with 95% CI for MDA achievement between obese or overweight patients with RA or PsA and normal-weight control subjects were included. Obesity and overweight were defined according to BMI categories of included patients: normal weight (BMI <25 kg/m^2^), overweight (BMI 25–30 kg/m^2^), and obese (BMI >30 kg/m^2^). MDA achievement was defined on the basis of Disease Activity Score in 28 joints (DAS28) or according to Coates criteria during treatment. From each study, data regarding sample size, major clinical and demographic variables, and data about MDA achievement were extracted. To exclude the risk of data overlap, original databases were analyzed for studies performed in the same institutions. Evaluation of methodological quality of each study was not performed, because it failed to demonstrate a clear advantage [21].

### Statistical analysis and risk of bias assessment

Statistical analysis was carried out using Comprehensive Meta-analysis version 2 software (2005; Biostat, Englewood, NJ, USA). Differences in MDA achievement between obese or overweight patients with RA or PsA and normal-weight control subjects were expressed as ORs with pertinent 95% CIs. The overall effect was tested using Z-scores, and significance was set at *p* < 0.05. Furthermore, meta-regression analysis was performed to evaluate the impact of clinical and demographic data on the evaluated outcomes.

Statistical heterogeneity between studies was assessed with chi-square test or Cochran’s Q test, and the *I* ^2^ statistic, which measures inconsistency across study results and describes the proportion of total variation in study estimates that is due to heterogeneity rather than sampling error. In detail, *I* ^2^ values of 0% indicate no heterogeneity, 25% low heterogeneity, 25–50% moderate heterogeneity, and 50% high heterogeneity [22]. Publication bias was represented graphically by funnel plots of the standard difference in means versus the standard error. Visual inspection of funnel plot asymmetry was performed to address possible small-study effect [23]. In order to be as conservative as possible, the random-effect method was used to take into account the variability among included studies.

### Subgroup analyses

We also planned to perform subanalyses of results according to the type of rheumatic disease evaluated (RA or PsA).

### Sensitivity analyses

Given the several confounding factors that might impact the difference in MDA achievement between obese or overweight patients and normal-weight control subjects, a sensitivity analysis was performed by pooling only ORs of MDA achievement obtained by means of multivariate analysis and adjusted for a series of potential confounders. In addition, a further sensitivity analysis was performed after excluding studies defining obesity as BMI >25 kg/m^2^ and the study defining MDA as an on-treatment DAS28 < 5.1.

### Meta-regression analyses

We hypothesized that differences among included studies might be affected by demographic (mean age, male sex) and clinical (follow-up duration, treatment duration, baseline DAS28, disease duration, C-reactive protein [CRP] and erythrocyte sedimentation rate [ESR] at baseline) variables. To assess the possible effect of such variables in explaining different results observed across studies, we planned to perform meta-regression analyses after implementing a regression model with MDA achievement as a dependent variable (*y*) and the above-mentioned covariates as independent variables (*x*). This analysis was performed with Comprehensive Meta-analysis version 2 software.

## Results

As reported in Additional file 1: Figure S1, of the 603 retrieved studies, 583 were excluded because they were reviews or case reports or were judged to be off the topic after scanning the title and/or the abstract. Another three studies were excluded after full-length paper evaluation because they reported only data about radiographic joint damage progression. Thus, 17 studies [7, 15–19, 24–34] (10 on RA and 7 on PsA) comprising a total of 6693 patients (1562 with PsA and 5131 with RA) were included in the analysis. All the included studies stratified the study population according to the presence of obesity. In addition, eight studies [17–19, 29–33] also reported data on overweight patients.

### Study characteristics

The major characteristics of included studies are shown in Table 1. With the exception of three studies in which researchers defined obesity as a BMI >25 kg/m^2^ [18, 27, 28], patients were categorized into the following groups according to their body mass index (BMI): normal (<25 kg/m^2^), overweight (25–30 kg/m^2^), and obese (>30 kg/m^2^). Overall, a total of 1765 (26.4%) of 6693 enrolled patients were obese, 1579 (23.6%) were overweight, and 3349 (50.0%) were normal weight.

**Table 1:** Characteristics of included studies

First author, year [reference]	Population	Number of subjects	Obese, *n* (%)	Overweight, *n* (%)	Male sex, %	Age, years	Disease duration, months	Therapy duration, months	CRP, mg/dl	ESR, mm/h
Ajeganova, 2013 [33]	RA	1333	211 (15.8)	524 (39.3)	30.8	64.6	–	–	0.4	13
Choe, 2014 [28]	RA	568	108 (19.0)	–	11.3	56.6	85.2	–	0.07	31.2
Di Minno, 2012 [3]	PsA	270	111 (41.1)	–	45.9	51.73	58.44	12	0.264	18.05
Di Minno, 2012 [6]	PsA	90	29 (32.2)	–	46.7	52.5	108.96	12	0.22	17.9
Di Minno, 2013 [15]	PsA	270	135 (50)	–	46	51.7	110.9	12	2.6	18.1
Di Minno, 2014 [5]	PsA	76	44 (57.9)	–	43.6	46.2	114	6	0.42	20.2
Eder, 2015 [19]	PsA	557	197 (35.4)	200 (35.9)	58.7	52.1	138	12	1.49	20.3
Ellerby, 2014 [25]	RA	233	57 (24.5)	–	30.9	60.8	129.6	36	–	–
Gremese, 2013 [29]	RA	641	66 (10.3)	207 (32.3)	18.7	52.1	100.8	12	–	35.9
Heimans, 2013 [27]	RA	508	292 (57.5)	–	32.6	54.7	–	12	2.06	35.7
Iannone, 2013 [17]	PsA	135	45 (33.3)	47 (34.8)	50.4	53.2	120	42.6	1.5	25.6
Iannone, 2015 [26]	RA	292	66 (22.6)	–	14.8	57.9	146.4	12	2,2	37.4
Iervolino, 2012 [30]	PsA	136	82 (60.3)	50 (36.8)	42.6	45.62	62.28	3	0.602	21.93
Klaasen, 2011 [31]	RA	89	15 (16.9)	66 (74.2)	25.9	55.7	98.9	4	2.27	34.7
Rodrigues, 2014 [34]	RA	317	73 (23.0)	–	–	–	–	6	–	–
Sandberg, 2014 [18]	RA	495	85 (17.2)	170 (34.3)	28.7	–	6	6	–	–
Westhoff, 2007 [32]	RA	767	149 (19.4)	315 (41.1)	28.4	57.2	11.5	36	2.15	–

*Abbreviations: RA* rheumatoid arthritis, *PsA* psoriatic arthritis, *CRP* C reactive protein, *ESR* erythrocyte sedimentation rate

The mean age of the subjects ranged from 45.6 to 64.6 years, the prevalence of male sex from 11.3% to 58.7%, and the treatment duration from 3 to 42.6 months. The cutoffs used to define achievement of MDA were based on an on-treatment DAS28 score <2.6 in seven studies [17, 18, 25, 28, 29, 33, 34], <5.1 in one study [32], or according to the Coates criteria in six studies [7, 15, 16, 19, 24, 30].

### MDA and body weight

**Fig. 1:**
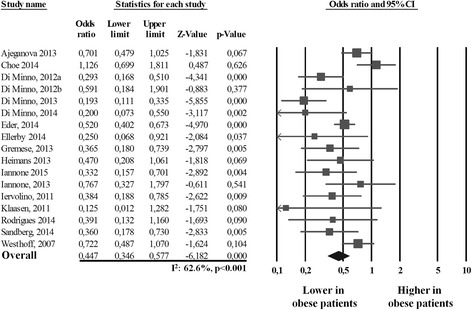
Forest plot of minimal disease activity achievement in obese patients versus normal-weight patients with rheumatic diseases (rheumatoid arthritis or psoriatic arthritis)

All the 17 studies [7, 15–19, 24–34] provided data about achievement of MDA in 1765 obese patients with PsA or RA receiving a treatment with traditional or biologic DMARDs. As showed in Fig. 1, the remission rate was significantly lower in the 1765 obese patients than in the 3349 normal-weight subjects (30.9%, [95% CI 21.1–42.8%] vs. 50.0% [95% CI 38.8–61.1%]) with a corresponding OR of 0.447 (95% CI 0.346–0.577, *p* < 0.001, *I* ^2^ = 62.6%, *p* < 0.001). The heterogeneity was not reduced by the exclusion of one study at a time. Overall, the risk of not achieving MDA attributable to the presence of obesity was 38.2%.

**Fig. 2:**
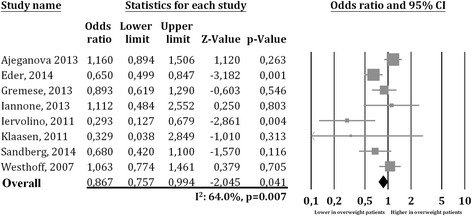
Forest plot of minimal disease activity achievement in overweight patients versus normal-weight patients with rheumatic diseases (rheumatoid arthritis or psoriatic arthritis)

Interestingly, a significant difference was also found in eight studies [17–19, 29–33] in which researchers reported data on overweight patients and showed a significantly lower prevalence of MDA achievement in the 1579 overweight patients (297 with PsA and 1282 with RA) than in the 1794 normal-weight subjects (OR 0.867, 95% CI 0.757–0.994, *p* = 0.041, *I* ^2^ = 64%, *p* = 0.007) (Fig. 2). The heterogeneity was not reduced by the exclusion of one study at a time.

### Subgroup analyses (PsA and RA)

As shown in Table 2, 10 of the 17 studies [18, 25–29, 31–34] included in our meta-analysis enrolled patients with RA, whereas the remaining 7 [7, 15–17, 19, 24, 30] examined patients with PsA. Overall, the studies on patients with RA comprised a total of 3849 patients, including 1122 obese subjects compared and 2727 normal-weight control subjects. The studies on PsA comprised a total of 1265 patients, including 643 obese subjects and 622 normal-weight control subjects. The MDA achievement rate was significantly higher in those who had a normal body weight than in obese patients, both in patients with RA (OR 0.583, 95% CI 0.401–0.848, *p* = 0.005) and in those with PsA (OR 0.369, 95% CI 0.249–0.546, *p* < 0.001). In contrast, when we evaluated the effect of overweight, we found that these results were confirmed for PsA but not for RA (Table 2).

**Table 2:** Odds of achieving minimal disease activity in obese patients versus normal-weight patients, stratified according to type of rheumatic disease (rheumatoid arthritis and psoriatic arthritis)

	Number of studies	Population, *n*	OR (95% CI), *p* value	Heterogeneity (*I* ^2^, *p* value)
Obesity vs. normal weight				
Rheumatoid arthritis	10		0.583 (0.401–0.848), *p* = 0.005	*I* ^2^ = 66.2%, *p* = 0.002
Obese		1122		
Normal weight		2727		
Psoriatic arthritis	7		0.369 (0.249–0.546), *p* < 0.001	*I* ^2^ = 63.1%, *p* = 0.012
Obese		643		
Normal weight		622		
Overweight vs. normal weight				
Rheumatoid arthritis	5		1.00 (0.849–1.182), *p* = 0.984	*I* ^2^ = 23.7%, *p* = 0.263
Overweight		1282		
Normal weight		1517		
Psoriatic arthritis	3		0.637 (0.500–0.811), *p* < 0.001	*I* ^2^ = 60.2%, *p* = 0.081
Overweight		297		
Normal weight		277		

### Sensitivity analyses

To minimize the impact of several confounding factors on the difference in MDA achievement between obese or overweight patients and normal-weight control subjects, we pooled data from the five studies [18, 19, 25, 27, 34] in which researchers reported adjusted risk estimates. This sensitivity analysis consistently confirmed the lower MDA achievement, both in obese (OR 0.481, 95% CI 0.385–0.602) and in overweight patients (OR 0.657, 95% CI 0.521–0.829), than in normal-weight control subjects. In addition, these results were confirmed after we excluded studies [18, 27, 28] defining obesity as BMI >25 kg/m^2^ and the study [32] defining MDA as an on-treatment DAS28 < 5.1 (Additional file 1: Figure S2).

#### Publication bias

Funnel plots of effect size versus standard error for studies evaluating the association of BMI with MDA rate were quite symmetrical, suggesting absence of publication bias and of small-study effect (Additional file 1: Figure S3). Egger’s test confirmed the absence of publication bias both for studies on obesity (*p* =0.285) and for studies on overweight (*p* = 0.314).

#### Meta-regression analysis

**Fig. 3:**
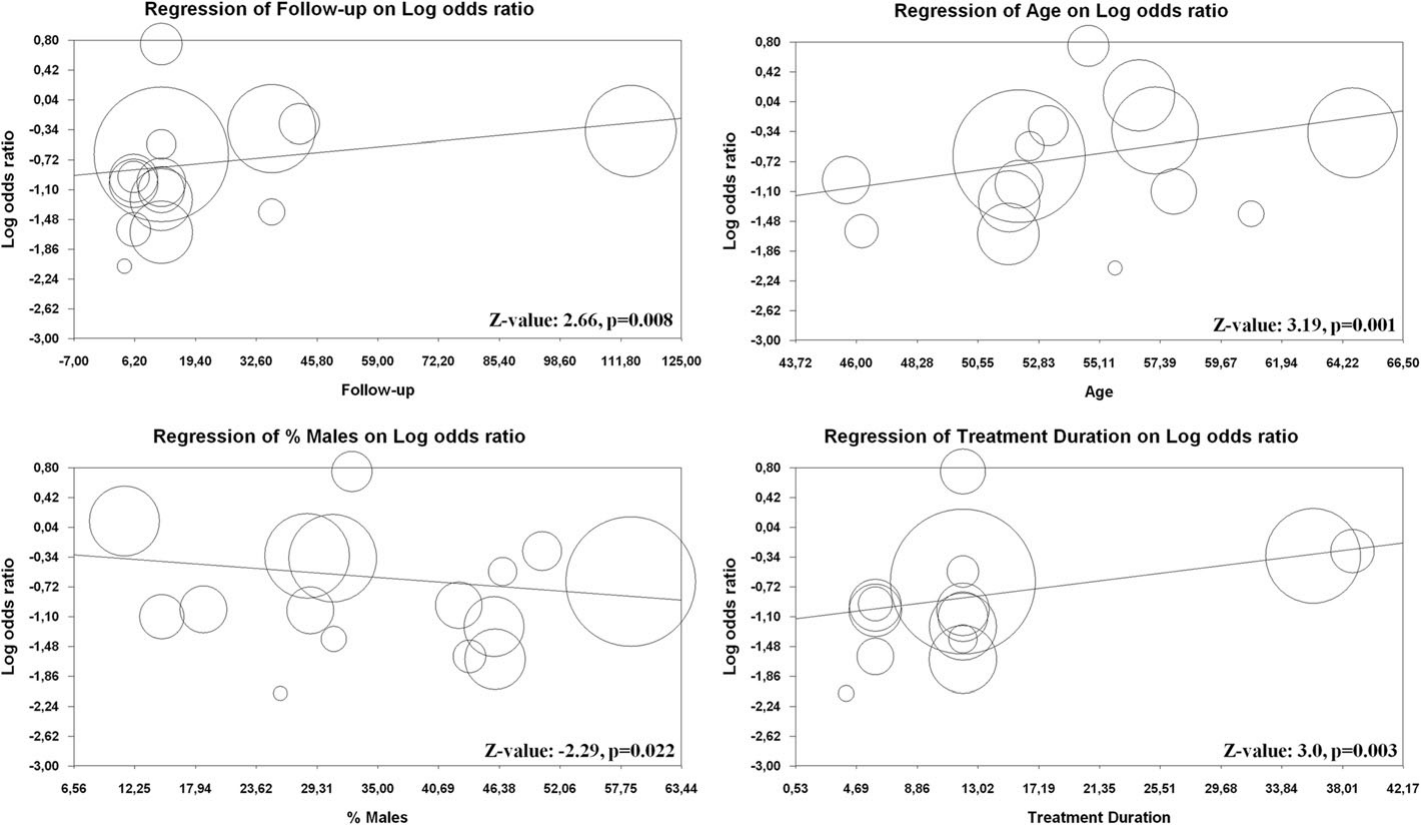
Meta-regression of the effect of follow-up duration, age, sex, and treatment duration on the difference in minimal disease activity achievement between obese patients and control subjects

To further assess the impact of BMI on decreasing chances of remission, some meta-regression analyses were performed. While follow-up, age, male sex, and treatment duration affected the difference in MDA achievement between obese or overweight patients and normal-weight subjects (Z-value 2.66, *p* = 0.008; Z-value 3.19, *p* = 0.001; Z-value −2.29, *p* = 0.022; Z-values 3.0, *p* = 0.003, respectively) (Fig. 3), baseline DAS28, disease duration, CRP, and ESR at baseline did not (Z-value −0.22, *p* = 0.827; Z-value −1.08, *p* = 0.277, Z-value −1.60, *p* = 0.108; and Z-value 1.62, *p* = 0.104, respectively) (Additional file 1: Figure S4).

## Discussion

In the present meta-analysis, we evaluated the impact of obesity or overweight on the achievement of MDA in patients with PsA and patients with RA receiving treatment with traditional or biologic DMARDs. We found a significantly lower MDA achievement in obese patients (BMI >30 kg/m^2^) than in those with a normal weight (BMI <25 kg/m^2^). Interestingly, a significant difference has been also documented in overweight patients (BMI from 25 to 30 kg/m^2^) compared with those with a normal weight (BMI <25 kg/m^2^). Moreover, all results were confirmed when we separately analyzed patients with RA and patients with PsA.

Meta-regression analyses refined and extended our findings. Interestingly, we found that follow-up, treatment duration, age, and male sex affected the difference in MDA achievement between obese patients and normal-weight subjects. In detail, we found that a progressive increase in treatment duration leads to a lower difference in the rate of MDA achievement between obese patients and those with a normal weight. This result might suggest that obese patients may need a longer-lasting antirheumatic treatment to achieve MDA. However, data about the time needed to achieve MDA are not available; thus, further studies with ad hoc designs are needed to verify this hypothesis.

With regard to age and sex, we found that the reduction in MDA achievement due to obesity is more significant in males and in younger subjects. This finding is of particular interest, considering that some previous data suggested that a young age and male sex are able to identify patients most likely to achieve MDA during treatment with antirheumatic drugs [30, 35]. Thus, according to our results, obesity could hamper the effect of these predictors of MDA achievement. However, these results could be due to chance, and further studies evaluating potential underlying mechanisms are needed to address this issue.

Several different mechanisms may be involved in the explanation of the impact of obesity on MDA achievement. It is known that adipose tissue has an endocrine function because it secretes many molecules (adipokines) involved in the inflammatory network. These mediators include mainly adiponectin, leptin, plasminogen activator inhibitor-1 (PAI-1), IL-6, and TNF-α. Adiponectin is an anti-inflammatory cytokine. A low concentration of adiponectin is associated with insulin resistance and impaired endothelium-dependent vasodilation. Low serum levels of adiponectin have been reported in several chronic diseases such as obesity and psoriasis. Both leptin and PAI-1 induce endothelial dysfunction. In addition, IL-6 and TNF-α promote insulin resistance in skeletal muscle and liver as well as proinflammatory activity in synovial tissue. Moreover, TNF-α induces endothelial dysfunction and liver synthesis of clotting factors, thus predisposing individuals to atherosclerosis and atherothrombosis [36]. High levels of TNF-α and IL-6 eventually reduce adiponectin production by adipose tissue, determining a self-maintained system.

A further relevant information that could be derived from our study is the prevalence of obesity in patients with rheumatic disease. Interestingly, patients were consecutively enrolled in the included studies, and among 6693 patients (1562 with PsA and 5131 with RA), the prevalence of obesity or overweight was about 50%. This finding further strengthens the hypothesis of the high prevalence of cardiometabolic risk factors in patients with rheumatic diseases [4, 37]. Much data in the literature supports this possibility [38–40], and the finding that the vascular morbidity/mortality of patients with rheumatic diseases resembles that of patients with type 2 diabetes mellitus further helps define the severity of the CV risk in this clinical setting [41]. In fact, obesity is associated with a hyperexpression of TNF-α and other adipokines (e.g., IL-6, leptin, adiponectin) and transforming growth factor-β, which leads to a chronic proinflammatory state. Moreover, subjects with rheumatic diseases exhibit an enhanced prevalence of the metabolic syndrome and of some of its major features (obesity, hypertension, hypercholesterolemia, hypertriglyceridemia, and impaired fasting glucose). However, such an association does not entirely explain the extent of premature atherosclerosis in subjects with rheumatic diseases, and inflammation appears to act synergistically with traditional vascular risk factors (VRFs). In fact, several studies demonstrated that patients with rheumatic diseases had increased platelet reactivity [6–8], a higher grade of subclinical atherosclerosis [4, 9], impairment of endothelial function, and arterial stiffness [10–12], as compared with control subjects matched for CV risk factors. In this setting, antirheumatic treatment was demonstrated to have beneficial effects on lowering the CV risk in these patients by controlling systemic inflammation, with maximum effect in those who achieved MDA [8].

Our study has some potential limitations. First, the studies included in our meta-analysis had different inclusion and exclusion criteria, and there was significant heterogeneity among the studies. Interestingly, all studies used standardized BMI cutoffs to define the presence/absence of obesity/overweight. However, three studies that defined obesity as a BMI >25 kg/m^2^ [18, 27, 28] did not provide separate data for overweight and obesity. Interestingly, when we repeated the analyses after excluding these three studies, all results were entirely confirmed. A further potential source of bias is the lack of data about changes in BMI during the study period. Some evidence in the literature suggested that treatment with antirheumatic drugs is able to modify BMI [3, 42] and that weight loss is able to increase the MDA achievement rate [5]. This should be taken into account when interpreting results of the present meta-analysis.

The definition of MDA was highly variable among studies. The cutoff to define MDA achievement was based on an on-treatment DAS28 score <2.6 in seven studies [17, 18, 25, 28, 29, 33, 34], <5.1 in one study [32], and according to the Coates criteria in six studies on PsA [7, 15, 16, 19, 24, 30]. In addition, both DAS28 and Coates criteria also include ESR and CRP values for the assessment of disease activity. Because obesity is associated with low-grade inflammation [13], ESR and CRP levels can result in elevated independently of disease activity. This may lead to a misclassification of MDA achievement. To overcome this problem, newer criteria for MDA definition, specific for obese patients and independent of ESR and CRP, should be used in future studies. Furthermore, there are some major differences in the prevalence of clinical and demographic data of enrolled patients, and, with the meta-regression approach, we were able to adjust results for some but not all potential confounders. Thus, extreme caution is necessary in interpretation of the overall results.

Although it was not possible to conclusively ascertain sources of heterogeneity, all results were confirmed in all subgroups and sensitivity analyses, and no publication bias was found in our analyses. Moreover, to take into account the effect of confounding factors on the difference in the MDA achievement between obese or overweight patients and normal-weight control subjects, we repeated the analysis by pooling only data adjusted for potential confounders, and all results were confirmed. Differences in the types of rheumatic disease studied might represent a potential source of bias. In particular, 10 of the 17 studies included in our meta-analysis were performed with patients with RA, whereas the remaining 7 studies were of patients with PsA. Interestingly, the results obtained in the primary analysis were entirely confirmed when we separately analyzed data on PsA and RA.

## Conclusions

Despite some limitations, the results of our study suggest that obesity and overweight reduce the chances to achieve MDA in patients with rheumatic diseases starting treatment with antirheumatic drugs.

## Additional file

Additional file 1: Online-only supplemental data (PRISMA flow diagram, publication bias and meta-regression analyses). (DOC 317 kb)

## Abbreviations

BMI: Body mass index
CRP: C-reactive protein
CV: Cardiovascular
DAS28: Disease Activity Score in 28 joints
DMARD: Disease-modifying antirheumatic drug
ESR: Erythrocyte sedimentation rate
IL-6: Interleukin-6
MDA: Minimal disease activity
PAI-1: Plasminogen activator inhibitor-1
PRISMA: Preferred Reporting Items for Systematic reviews and Meta-Analyses
PsA: Psoriatic arthritis
RA: Rheumatoid arthritis
TNF-α: Tumor necrosis factor α
VRF: Vascular risk factor

## References

[1] Alamanos Y, Drosos AA (2005). Epidemiology of adult rheumatoid arthritis. Autoimmun Rev.

[2] Taylor W, Gladman D, Helliwell P, Marchesoni A, Mease P, Mielants H, CASPAR Study Group (2006). Classification criteria for psoriatic arthritis: development of new criteria from a large international study. Arthritis Rheum.

[3] Di Minno MN, Iervolino S, Lupoli R, Russolillo A, Coppola A, Peluso R (2012). Cardiovascular risk in rheumatic patients: the link between inflammation and atherothrombosis. Semin Thromb Hemost.

[4] Di Minno MN, Ambrosino P, Lupoli R, Di Minno A, Tasso M, Peluso R (2015). Cardiovascular risk markers in patients with psoriatic arthritis: a meta-analysis of literature studies. Ann Med.

[5] Di Minno MN, Peluso R, Iervolino S, Russolillo A, Lupoli R, Scarpa R, CaRRDs Study Group (2014). Weight loss and achievement of minimal disease activity in patients with psoriatic arthritis starting treatment with tumor necrosis factor α blockers. Ann Rheum Dis.

[6] Di Minno MN, Iervolino S, Peluso R, Scarpa R, Di Minno G (2012). Platelet reactivity and disease activity in subjects with psoriatic arthritis. J Rheumatol.

[7] Di Minno MN, Iervolino S, Peluso R, Di Minno A, Ambrosino P, Scarpa R, CaRRDs Study Group (2014). Hemostatic and fibrinolytic changes are related to inflammatory conditions in patients with psoriatic arthritis—effect of different treatments. J Rheumatol.

[8] Di Minno MN, Iervolino S, Zincarelli C, Lupoli R, Ambrosino P, Pizzicato P (2015). Cardiovascular effects of etanercept in patients with psoriatic arthritis: evidence from the cardiovascular risk in rheumatic diseases database. Expert Opin Drug Saf.

[9] Ambrosino P, Lupoli R, Di Minno A, Tasso M, Peluso R, Di Minno MN (2015). Subclinical atherosclerosis in patients with rheumatoid arthritis: a meta-analysis of literature studies. Thromb Haemost.

[10] Di Minno MN, Iervolino S, Peluso R, Scarpa R, Di Minno G, CaRRDs Study Group (2011). Carotid intima-media thickness in psoriatic arthritis: differences between tumor necrosis factor-α blockers and traditional disease-modifying antirheumatic drugs. Arterioscler Thromb Vasc Biol.

[11] Di Minno MN, Ambrosino P, Lupoli R, Di Minno A, Tasso M, Peluso R (2015). Clinical assessment of endothelial function in patients with rheumatoid arthritis: a meta-analysis of literature studies. Eur J Intern Med.

[12] Ambrosino P, Tasso M, Lupoli R, Di Minno A, Baldassarre D, Tremoli E (2015). Non-invasive assessment of arterial stiffness in patients with rheumatoid arthritis: a systematic review and meta-analysis of literature studies. Ann Med.

[13] Russolillo A, Iervolino S, Peluso R, Lupoli R, Di Minno A, Pappone N (2013). Obesity and psoriatic arthritis: from pathogenesis to clinical outcome and management. Rheumatology (Oxford).

[14] Di Minno MN, Ambrosino P, Peluso R, Di Minno A, Lupoli R, Dentali F, CaRRDs Study Group (2014). Lipid profile changes in patients with rheumatic diseases receiving a treatment with TNF-α blockers: a meta-analysis of prospective studies. Ann Med.

[15] Di Minno MN, Peluso R, Iervolino S, Lupoli R, Russolillo A, Scarpa R (2013). Obesity and the prediction of minimal disease activity: a prospective study in psoriatic arthritis. Arthritis Care Res (Hoboken).

[16] Di Minno MN, Peluso R, Iervolino S, Lupoli R, Russolillo A, Tarantino G (2012). Hepatic steatosis, carotid plaques and achieving MDA in psoriatic arthritis patients starting TNF-α blockers treatment: a prospective study. Arthritis Res Ther.

[17] Iannone F, Fanizzi R, Scioscia C, Anelli MG, Lapadula G (2013). Body mass does not affect the remission of psoriatic arthritis patients on anti-TNF-α therapy. Scand J Rheumatol.

[18] Sandberg ME, Bengtsson C, Källberg H, Wesley A, Klareskog L, Alfredsson L (2014). Overweight decreases the chance of achieving good response and low disease activity in early rheumatoid arthritis. Ann Rheum Dis.

[19] Eder L, Thavaneswaran A, Chandran V, Cook RJ, Gladman DD (2015). Obesity is associated with a lower probability of achieving sustained minimal disease activity state among patients with psoriatic arthritis. Ann Rheum Dis.

[20] Moher D, Liberati A, Tetzlaff J, Altman DG (2009). Preferred reporting items for systematic reviews and meta-analyses: the PRISMA statement. PLoS Med.

[21] Juni P, Witschi A, Bloch R, Egger M (1999). The hazards of scoring the quality of clinical trials for meta-analysis. JAMA.

[22] Higgins JP, Thompson SG, Deeks JJ, Altman DG (2003). Measuring inconsistency in meta-analyses. BMJ.

[23] Sterne JA, Egger M, Smith GD (2001). Systematic reviews in health care: investigating and dealing with publication and other biases in meta-analysis. BMJ.

[24] Di Minno MN, Iervolino S, Peluso R, Russolillo A, Lupoli R, Scarpa R (2012). Hepatic steatosis and disease activity in subjects with psoriatic arthritis receiving tumor necrosis factor-α blockers. J Rheumatol.

[25] Ellerby N, Mattey DL, Packham J, Dawes P, Hider SL (2014). Obesity and comorbidity are independently associated with a failure to achieve remission in patients with established rheumatoid arthritis. Ann Rheum Dis.

[26] Iannone F, Fanizzi R, Notarnicola A, Scioscia C, Anelli MG, Lapadula G (2015). Obesity reduces the drug survival of second line biological drugs following a first TNF-α inhibitor in rheumatoid arthritis patients. Joint Bone Spine.

[27] Heimans L, van den Broek M, le Cessie S, Siegerink B, Riyazi N, Han KH (2013). Association of high body mass index with decreased treatment response to combination therapy in recent-onset rheumatoid arthritis patients. Arthritis Care Res (Hoboken).

[28] Choe JY, Bae J, Lee H, Park SH, Kim SK (2014). Lack association of body mass index with disease activity composites of rheumatoid arthritis in Korean population: cross-sectional observation. Clin Rheumatol.

[29] Gremese E, Carletto A, Padovan M, Atzeni F, Raffeiner B, Giardina AR (2013). Obesity and reduction of the response rate to anti-tumor necrosis factor α in rheumatoid arthritis: an approach to a personalized medicine. Arthritis Care Res (Hoboken).

[30] Iervolino S, Di Minno MN, Peluso R, Lofrano M, Russolillo A, Di Minno G (2012). Predictors of early minimal disease activity in patients with psoriatic arthritis treated with tumor necrosis factor-α blockers. J Rheumatol.

[31] Klaasen R, Wijbrandts CA, Gerlag DM, Tak PP (2011). Body mass index and clinical response to infliximab in rheumatoid arthritis. Arthritis Rheum.

[32] Westhoff G, Rau R, Zink A (2007). Radiographic joint damage in early rheumatoid arthritis is highly dependent on body mass index. Arthritis Rheum.

[33] Ajeganova S, Andersson ML, Hafström I, BARFOT Study Group (2013). Association of obesity with worse disease severity in rheumatoid arthritis as well as with comorbidities: a long-term followup from disease onset. Arthritis Care Res (Hoboken).

[34] Rodrigues AM, Reis JE, Santos C, Pereira MP, Loureiro C, Martins F (2014). Obesity is a risk factor for worse treatment response in rheumatoid arthritis patients - results from reuma.pt [abstract A1.1]. Ann Rheum Dis.

[35] Glintborg B, Østergaard M, Dreyer L, Krogh NS, Tarp U, Hansen MS (2011). Treatment response, drug survival, and predictors thereof in 764 patients with psoriatic arthritis treated with anti-tumor necrosis factor α therapy: results from the nationwide Danish DANBIO registry. Arthritis Rheum.

[36] Di Minno MN, Tufano A, Ageno W, Prandoni P, Di Minno G (2012). Identifying high-risk individuals for cardiovascular disease: similarities between venous and arterial thrombosis in perspective. A 2011 update. Intern Emerg Med.

[37] Jamnitski A, Symmons D, Peters MJ, Sattar N, McInnes I, Nurmohamed MT (2013). Cardiovascular comorbidities in patients with psoriatic arthritis: a systematic review. Ann Rheum Dis.

[38] Jamnitski A, Visman IM, Peters MJ, Boers M, Dijkmans BA, Nurmohamed MT (2011). Prevalence of cardiovascular diseases in psoriatic arthritis resembles that of rheumatoid arthritis. Ann Rheum Dis.

[39] Han C, Robinson DW, Hackett MV, Paramore LC, Fraeman KH, Bala MV (2006). Cardiovascular disease and risk factors in patients with rheumatoid arthritis, psoriatic arthritis, and ankylosing spondylitis. J Rheumatol.

[40] Gladman DD, Ang M, Su L, Tom BD, Schentag CT, Farewell VT (2009). Cardiovascular morbidity in psoriatic arthritis. Ann Rheum Dis.

[41] Peters MJ, van Halm VP, Voskuyl AE, Smulders YM, Boers M, Lems WF (2009). Does rheumatoid arthritis equal diabetes mellitus as an independent risk factor for cardiovascular disease? A prospective study. Arthritis Rheum.

[42] Innala L, Möller B, Ljung L, Magnusson S, Smedby T, Södergren A (2011). Cardiovascular events in early RA are a result of inflammatory burden and traditional risk factors: a five year prospective study. Arthritis Res Ther.

