# Supplementary material for: Impact of body weight on the achievement of minimal disease activity in patients with rheumatic diseases: a systematic review and meta-analysis
Source: Arthritis Res Ther. 2016 Dec 13;18:297. doi: 10.1186/s13075-016-1194-8 (PMC5155390; doi:10.1186/s13075-016-1194-8)
Supplement: Additional file 1: — Online-only supplemental data (PRISMA flow diagram, publication bias and meta-regression analyses). (DOC 317 kb) [file 13075_2016_1194_MOESM1_ESM.doc]

**Impact of body weight on the achievement of minimal disease activity in patients with rheumatic diseases: a systematic review and meta-analysis.**

Roberta Lupoli, Paolo Pizzicato, Antonella Scalera, Pasquale Ambrosino, Manuela Amato,

Rosario Peluso, Matteo Nicola Dario Di Minno

Table of content:

| Figure S1 | Prisma Flow Diagram. |
| --- | --- |
| Figure S2 | Sensitivity analysis. Effect of obesity (Panel A) and overweight (Panel B) on the achievement of MDA after excluding studies defining obesity as Body Mass Index (BMI) > 25 kg/m2 and the study defining MDA as DAS-28 <5.1 |
| Figure S3 | Funnel plots of effect size versus standard error for studies evaluating the effect of obesity (Panel A) and overweight (Panel B) on the achievement of MDA. |
| Figure S4 | Meta-regression of the effect of DAS-28 at baseline, Disease duration, CRP and ESR on the difference in minimal disease activity achievement between obese patients and controls. |

**Figure S1.** **Prisma Flow Diagram**

identificatine

**Identification**

**Screening**

**Eligibility**

**Included**

Records identified through

database searching

(n = 603)

Records screened

(n = 603)

Records excluded

(n = 583)

Full-text articles assessed for eligibility

(n =20)

Full-text articles excluded with reasons

(n = 3)

- Data on radiographic joint damage progression

Studies included in quantitative synthesis (meta-analysis)

(n =17)

**Figure S2. Effect of obesity (Panel A) and overweight (Panel B) on the achievement of Minimal Disease Activity (MDA) in rheumatic patients after excluding studies defining obesity as Body Mass Index (BMI) > 25 kg/m^2^ and the study defining MDA as DAS-28 <5.1**

**Panel A**

**Panel B**

**Figure S3. Funnel plots of effect size versus standard error for studies evaluating the effect of obesity (Panel A) and overweight (Panel B) on the achievement of MDA.**

**Panel A: Obesity**

**
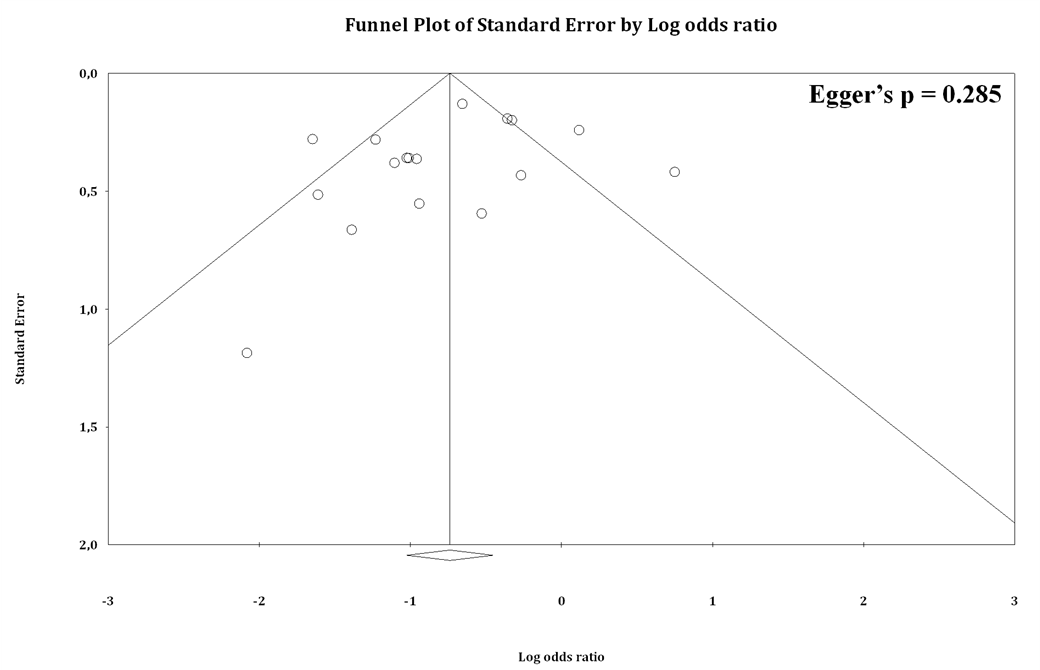
**

**Panel B: Overweight**

**
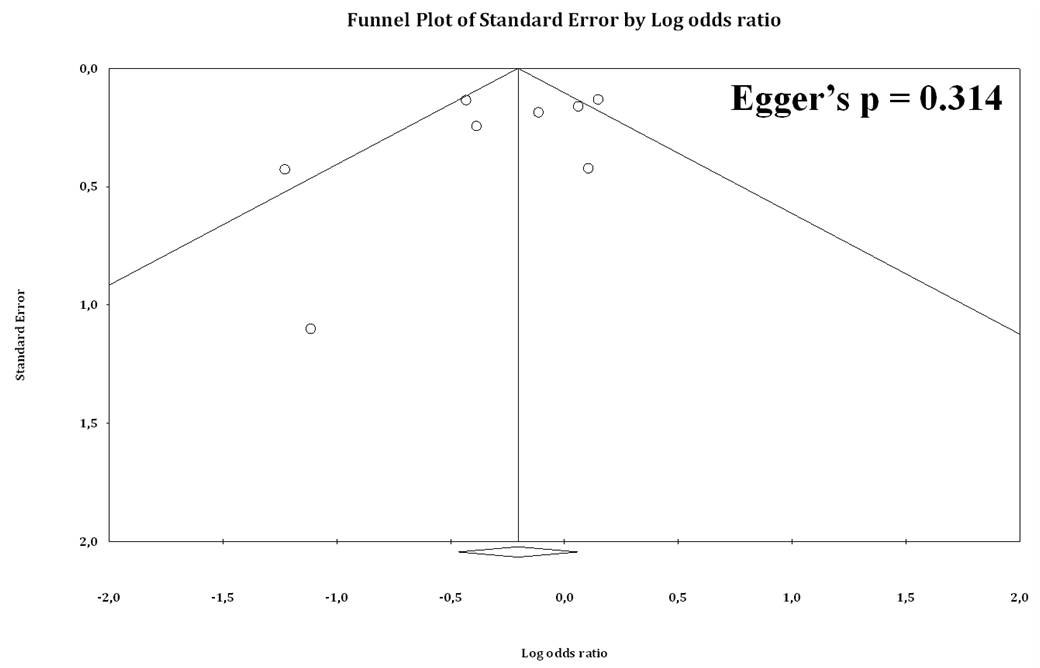
**

**Figure S4. Meta-regression of the effect of DAS-28 at baseline, Disease duration, CRP and ESR on the difference in minimal disease activity achievement between obese patients and controls.**

**
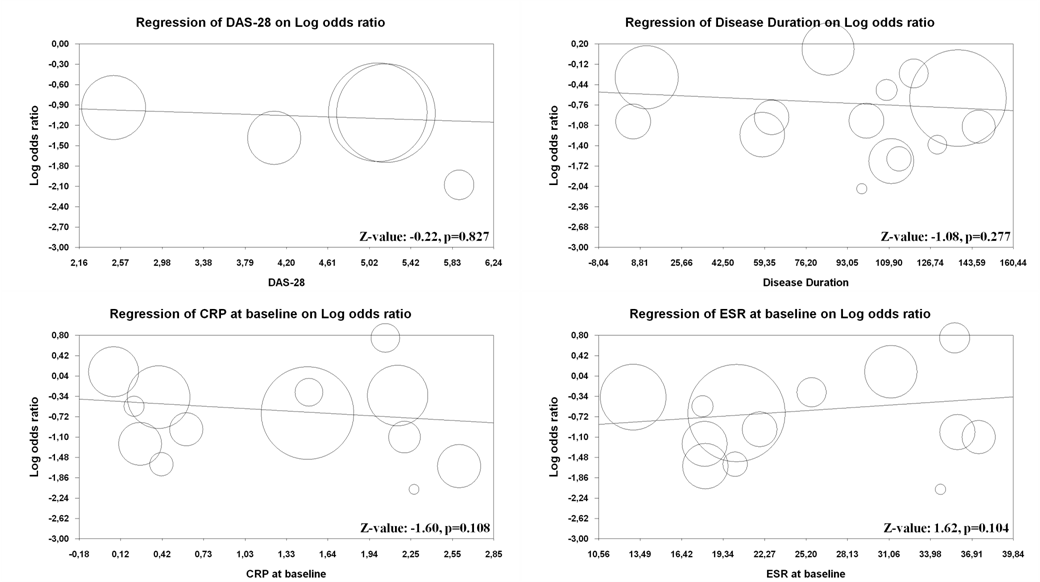
**
